# Investigating Interventions in Alzheimer's Disease with Computer Simulation Models

**DOI:** 10.1371/journal.pone.0073631

**Published:** 2013-09-16

**Authors:** Carole J. Proctor, Delphine Boche, Douglas A. Gray, James A. R. Nicoll

**Affiliations:** 1 Institute for Ageing and Health, Newcastle University, Newcastle upon Tyne, United Kingdom; 2 Clinical Neurosciences, Clinical and Experimental Sciences, University of Southampton, Southampton, United Kingdom; 3 Ottawa Hospital Research Institute, Ottawa, Canada; 4 Department of Biochemistry, Microbiology and Immunology, University of Ottawa, Ottawa, Canada; University of S. Florida College of Medicine, United States of America

## Abstract

Progress in the development of therapeutic interventions to treat or slow the progression of Alzheimer's disease has been hampered by lack of efficacy and unforeseen side effects in human clinical trials. This setback highlights the need for new approaches for pre-clinical testing of possible interventions. Systems modelling is becoming increasingly recognised as a valuable tool for investigating molecular and cellular mechanisms involved in ageing and age-related diseases. However, there is still a lack of awareness of modelling approaches in many areas of biomedical research. We previously developed a stochastic computer model to examine some of the key pathways involved in the aggregation of amyloid-beta (Aβ) and the micro-tubular binding protein tau. Here we show how we extended this model to include the main processes involved in passive and active immunisation against Aβ and then demonstrate the effects of this intervention on soluble Aβ, plaques, phosphorylated tau and tangles. The model predicts that immunisation leads to clearance of plaques but only results in small reductions in levels of soluble Aβ, phosphorylated tau and tangles. The behaviour of this model is supported by neuropathological observations in Alzheimer patients immunised against Aβ. Since, soluble Aβ, phosphorylated tau and tangles more closely correlate with cognitive decline than plaques, our model suggests that immunotherapy against Aβ may not be effective unless it is performed very early in the disease process or combined with other therapies.

## Introduction

Alzheimer's disease (AD) is characterised by aggregation of Aβ and tau proteins in the brain accompanied by glial cell activation together with synaptic and neuronal loss (reviewed in [1]). There is still no effective treatment which targets the underlying neurodegeneration in AD although many interventions are currently being tested. For example, a phase 11a clinical trial of PBT2, a metal-protein attenuating compound, has shown preliminary promising results although longer and larger trials are needed [2]. Based on the amyloid cascade hypothesis and promising experimental studies [3], some clinical trials of Aβ immunotherapy have shown hints of slowed deterioration in cognitive function [4], [5]. Unfortunately a small proportion of subjects treated with the first Aβ immunotherapy agent (AN1792) [6] developed aseptic meningoencephalitis and the trial had to be halted [7] and side effects remain a problem with agents currently in trials [8]. These side effects were unexpected and were not predicted by the pre-clinical animal models, demonstrating that animal models of AD do not replicate the complexity of the human disease [9]. Despite this serious drawback, animal models continue to be used extensively in the search for new therapies. We suggest that there is now a great need to develop new approaches to investigate possible new interventions. Mathematical modelling and computer simulation are relatively new approaches in the medical sciences but their potential as a useful complementary tool is being increasingly recognised.

Despite the problems associated with the clinical trials, a wealth of valuable data has been obtained and detailed analysis has been carried out. For example, neuropathological studies of patients with AD who were immunised against Aβ have shown that a reduction in Aβ plaques occurs [10]–[13] and this observation has subsequently been confirmed in vivo by amyloid imaging [14]. The actual mechanisms of how immunisation clears Aβ are not fully known but seem to involve phagocytosis of Aβ by microglia [12], [13], [15] and solubilisation of Aβ by antibody binding [10], [16]. In support of the amyloid hypothesis, immunotherapy-mediated removal of Aβ is accompanied by a reduction in phospho-tau [17], [18] and of a kinase putatively involved in mediating tau phosphorylation (GSK3β) [19]. The analysis of this data has provided the motivation to modify our previous dynamical model of the molecular mechanisms involved in the initiation and progression of AD [20] by including processes involved in Aβ immunisation.

As the pathways are complex and many of the mechanisms are not yet fully understood, it is necessary to make assumptions when building the model. There is also controversy over different mechanisms. This means that our assumptions will not be in accord with everyone's point of view.

For example, plaques are considered to be neuroprotective or having detrimental effects. We assume that Aβ can be detrimental by inducing production of reactive oxygen species (ROS), either as fibrillar (plaque) or soluble Aβ [21], [22]. Then ROS increase the production of p53 which leads to increased activity of GSK3β, increased phosphorylation of tau and hence the formation of tangles. Aβ may also directly enhance p53 production [23]. We also assume that soluble Aβ may inhibit the proteasome which would have detrimental effects on cells [24]. The formation of plaques decreases the pool of soluble Aβ and so prevents all these adverse effects, and is included as a neuroprotective role for plaques in our model.

Mathematical models of Aβ aggregation have been previously developed (e.g. [25],[26])and one in particular has been used to test interventions [27]. However, all these models have focussed only on Aβ and have not considered the role of tau aggregation which many consider to be more important than Aβ in the disease process [28]–[31]. The mechanisms which link Aβ and tau are still not fully known although many suggestions have been made [32]–[35]. The novelty of our model resides in that it seeks to not only examine the effect of interventions on Aβ but also how antibodies against Aβ can reduce tau pathology. In order to do this, we include possible players such as GSK3β, p53 and ROS that may link these two seemingly independent aggregation pathways as described in our previous mathematical model [20].

Since many of the mechanisms involved in the protein aggregation process are inherently random, and the numbers of molecules involved in many of the reactions we consider are small, it is more appropriate to use stochastic simulation for our models. However, the disadvantage of stochastic simulation is that it is very computer intensive and so we also use deterministic simulators for examining the effects of different parameters in the model. The models are represented as biochemical networks and are encoded in the widely used Systems Biology Markup Language (SBML) [36], [37]. This allows the models to be used in many different software tools and also allows easy modification as new data and hypotheses emerge. The models are freely available and provide a useful resource for researchers seeking new approaches in investigating the molecular mechanisms involved in AD and to test possible interventions.

## Results

### AD model and effect of clearance rate of Aβ monomers on plaque and tangle formation

**Figure 1:**
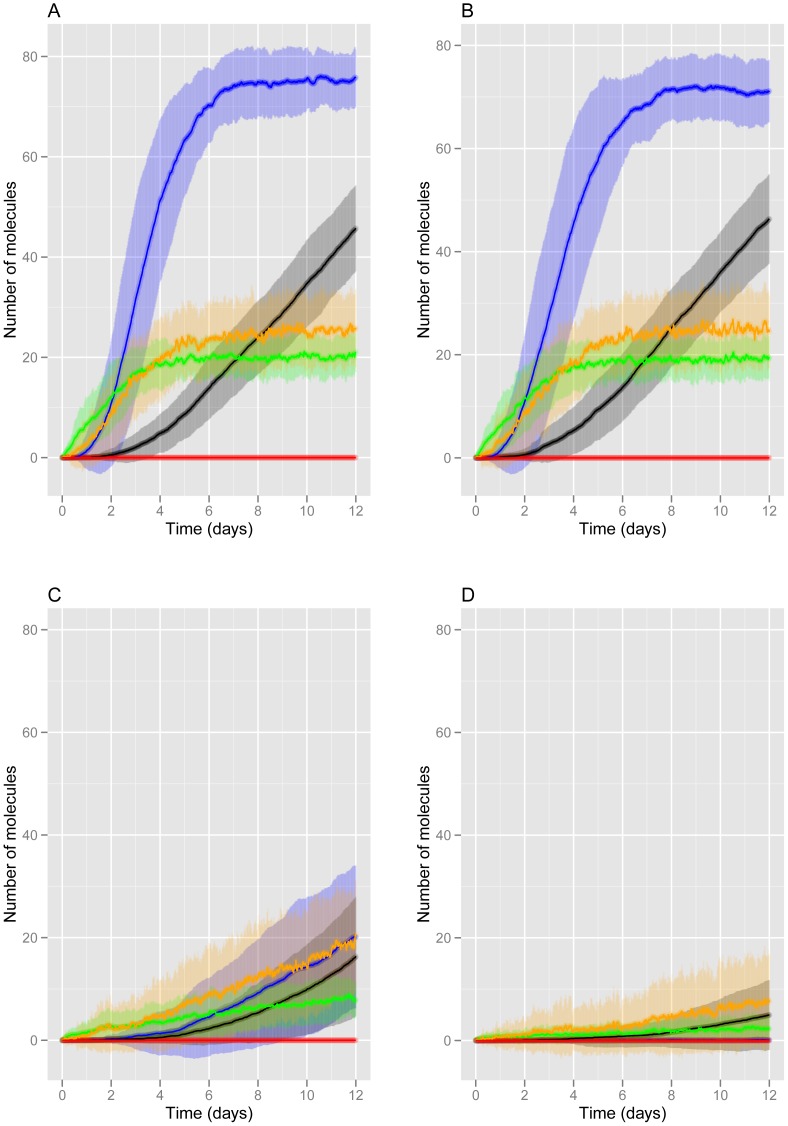
Model predictions for levels of Aβ and tau under normal and AD conditions (no immunisation). The mean curves from 100 stochastic simulations are plotted for different Aβ clearance rates. The shaded regions represent the one standard deviation from the mean. A *k_degAbeta_* = 1.5e-5 s^−1^), B *k_degAbeta_* = 2.1e-5 s^−1^), C *k_degAbeta_* = 1.0e-4s^−1^, D *k_degAbeta_* = 2.0e-4s^−1^. Key: orange = soluble Aβ; blue = Aβ plaques; green = phospho-tau; black = tau tangles; red = activated glia.

We used the model to mimic a cellular system whereby soluble Aβ is added to cells and the kinetics of its aggregation are followed over time scales of days. Therefore we parameterised our model using data from a cellular system [38]and carried out all the simulations over a 12 day period. In the experimental setting, after addition of Aβ to cells, aggregates form rapidly and are usually observed within 24 hours and reach maximum levels by about 2 days. In our computer model, the level of Aβ is initially zero, then monomers are produced and if the production rate is greater than the degradation rate, the monomer levels increase, form dimers and may start to aggregate as plaques. The rates for Aβ production and clearance are based on experimental data from a study showing that the Aβ production rate was the same in AD subjects and age-matched controls (1.86e^−5^ molecules s^−1^) but that the degradation rate was lower in the AD subjects (1.5e-5s^−1^) compared to control subjects (2.1e-5s^−1^) [39]. This means that the ratio of Aβ production to Aβ degradation is about 1.24 and 0.89 for AD and control subjects respectively, so the ratio of production to degradation is greater than one in the AD subjects which explains why Aβ levels increase over time. The experimental data was based on measurements in the cerebral spinal fluid rather than brain. Although this may be a limitation in the model, it is the relative rates of production and degradation that are important rather than their absolute rates since Aβ only accumulates if production is greater than degradation. The Aβ production and degradation rates of the AD subjects was used in our model. We also tried the normal degradation rate to investigate if our model would predict less occurrence of plaques. However, since we have accelerated the aggregation process for a cellular system as opposed to an ageing human brain, we found that we needed a much higher degradation rate for Aβ than that observed in normal aged brain [39] in order to prevent high levels of plaques forming under normal conditions (Figure 1). This is due to the stochastic simulation used and even if the production rate was lower than the degradation rate, occasional monomers will be present with the possibility of starting the aggregation process. If we use a degradation rate high enough, the model predicts that plaques never form over a 12 day period (Figure 1D). Using the lower degradation rate for AD, our model predicts a maximum plaque size of about 75 molecules reached in 6 days (from the time of formation) (Figure 1A). Figure 1 show the mean ± one standard deviation from the mean and it can be seen that there is variation in the levels of plaques and tangles, but the differences are not large (see also Figure S1 for plots of six individual stochastic simulations). It has been shown that there is high variability in the level of amyloid plaque content in AD patients with plaques even being undetected by imaging in some patients [40]. However, we should point out that by definition a patient cannot have AD without amyloid plaques and so this data may not be valid. The model presented here suggests that stochastic effects alone cannot explain the observed variability. However, the speeding up of the aggregation process, to mimic a cellular system, may have masked some of the variability as in a previous model of plaque formation examined over a period of 100 years, there was a very large variation in the age at which plaques first appeared [41]. Alternatively there may be effects due to other important factors such as genetic disposition which are currently missing from the model.

### Simulation of passive immunisation for the treatment of Alzheimer's disease

**Figure 2:**
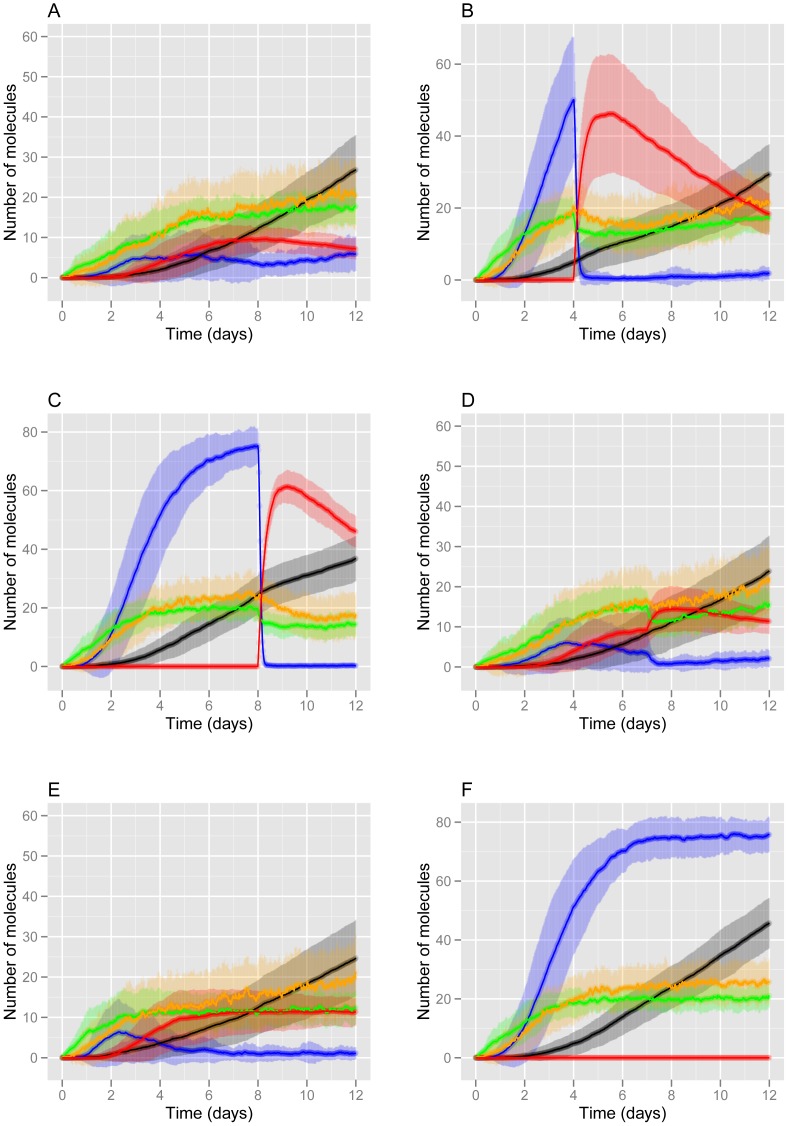
Model predictions for levels of Aβ, tau, activated glia under different simulated interventions. The mean curves from 100 stochastic simulations are plotted (six individual runs are shown in Fig. S2). The shaded regions represent the one standard deviation from the mean. A–D Simulated passive immunisation administered at different time-points: A Day 0; B Day 4; C Day 8; D Repeated immunisation at Day 0 and Day 7. E Simulated active immunisation. F No immunisation. Key: orange = soluble Aβ; blue = Aβ plaques; green = phospho-tau; black = tau tangles; red = activated glia.

For these simulations, the low degradation rate of Aβ (i.e. the AD rate) was used, so that plaques would form by day 4. In order to examine the effects of immunisation in the low degradation rate model, we simulated passive immunisation by adding the antibodies. The exact mechanisms by which antibodies work is still not clear and so we modelled three possible effects: enhanced degradation of soluble Aβ, enhanced disaggregation of plaques and activation of microglia to engulf and phagocytose plaques [15]. We simulated immunisation by using an event structure in the SBML code, whereby the species “antiAb” representing antibodies was set to 50 (the initial value being zero) at a specific time-point after the beginning of the simulation. If immunisation is simulated at day 4, when levels of plaques are low prior to the treatment, then the model predicts that levels of plaques are reduced and that initially there is a reduction in soluble Aβ. However following the disaggregation of plaques, soluble Aβ levels rise again and levels of plaques start to slowly increase (Figure 2B). The model also predicts a decrease in levels of phospho tau immediately after immunisation on day 4 and although levels of phospho tau continue to increase with time, this occurs at a much slower rate than prior to immunisation (green curve, Figure 2B). The reduction in levels of phospho tau by immunisation agrees with neuropathological data [10]. If immunisation is delayed until day 8, then levels of plaques are almost at their maximum prior to treatment, and so the increase of microglial activation is much more rapid and there is consequently a rapid clearance of plaques (Figure 2C). However, plaques are not totally cleared and remain above basal levels as a result of increased ROS in the system. There is no significant difference in the kinetics of tau phosphorylation or aggregation. Repeated interventions at day 0 and day 7 prevent plaques reaching high levels (Figure 2D). However, there is more activated microglia which may be detrimental due to their effects on ROS. Therefore the model suggests that an additional intervention to reduce ROS may be required if repeated immunisations are carried out. The model predicts that there is slightly slower kinetics in tau phosphorylation and aggregation with repeated interventions. We hypothesise that this is due to decreased activation of p53 and GSK3β via ROS which we confirmed by examining the level of active pools of p53 and GSK3β in the simulation output (data not shown). The model predicts some cell to cell variation in the model output as shown by the shaded areas in Figure 2 which represent one standard deviation from the mean. For example, the regions representing the mean± one standard deviation for soluble Aβ after immunization at Day 4 (Figure 2B) compared to no immunization (Figure 2F) are very similar. This indicates that for a particular cell, the levels of soluble Aβ could be higher after immunisation than without immunisation. This stochastic variation may explain the biological variability in responses to immunotherapy [16] and why some immunised AD patients show increased soluble Aβ compared to AD controls [42].

### Simulation of active Aβ immunisation for the treatment of Alzheimer's disease

We chose to mimic the action of full length Aβ42 (AN1792) for the active immunisation. We assume that this treatment causes antibody levels to slowly increase and then remain elevated. We modelled this by assuming that the species “antiAb” is continually produced and included a reaction for antiAb production. The model predicts similar results to repeated passive immunisation (Figure 2E). The results for no immunisation are also shown (Figure 2F) for comparison.

### Simulation of Aβ immunisation for the prevention of Alzheimer's disease

In order to examine the effects of the possibility of using Aβ immunisation for the prevention of Alzheimer's disease, we simulated the addition of antibodies (simulating passive immunisation) at the start at the simulation (before any plaques had appeared). To do this we set the initial value of antiAb to equal 50. The model predicts that immunisation at day 0 dramatically reduced plaques over a 12 day period but had only a small effect on levels of soluble Aβ (orange curve, Figure 2A). This intervention also predicts slightly lower levels of phospho-tau (compare green curves in Figure 2A and 2F) in agreement with neuropathological data [10]. This prediction is due to less Aβ-induced ROS production and so less activation of p53, which based on biochemical data demonstrating physical association of p53 with GSK3β and an increase of GSK3β activity in a p53 concentration-dependent manner [43] may result in a decrease in GSK3β activity.

### Effects of immunisation on GSK3β and phospho-tau

**Figure 3:**
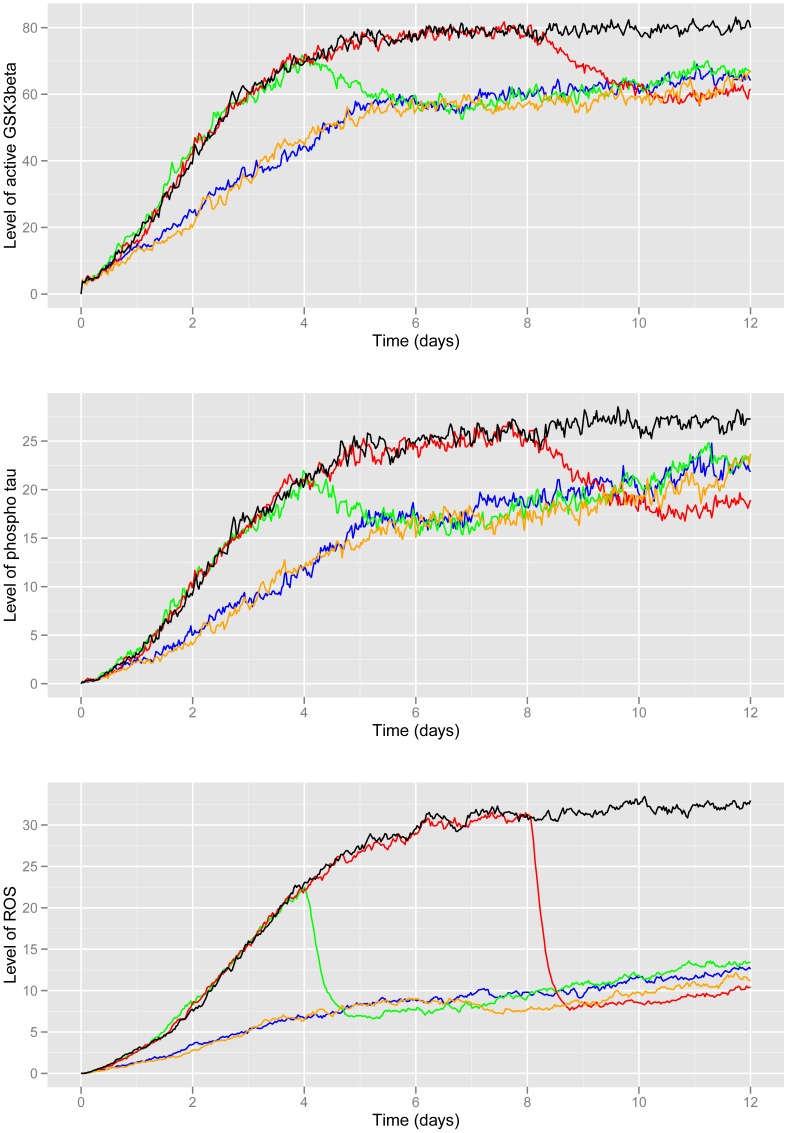
Model predictions for levels of ROS, Gsk3β and phospho-tau under different simulated interventions. The mean curves from 100 stochastic simulations are plotted. A Active Gsk3β B Phospho-tau; C ROS. Key: blue = day 0; green = day 4; red = day 8; D orange = repeated immunisation at Day 0 and Day 7; black = no immunisation.

Following Aβ immunisation we observed a lower GSK3β and a lower phospho-tau load in AD immunized patients [10], [17], [19]. Therefore we examined the model predictions on the levels of GSK3β and phospho-tau under the different interventions. As we currently do not include GSK3β turnover in our model (there being no evidence for the regulation of GSK3β at this level), we examined the levels of active GSK3β since phosphorylation of tau is dependent on this pool. The model predicts that interventions which start at day 0 lead to a delay in the increase in GSK3β activity (Figure 3A) but by day 12 there is very little difference in active GSK3β pools compared to later interventions. However, if the immunisation is repeated at day 7, the level of active GSK3β is lower throughout the 12 day period. The intervention at day 4 and day 8 show an immediate decrease in active pools of GSK3β after the simulated immunisation (green and red curves in Figure 3A). This is due to levels of plaques being fairly high prior to the immunisation so that microglia are activated, Aβ is cleared and ROS levels also decline (Figure 2 and 3C). The reduction in active GSK3β also leads to a reduction in phospho-tau (Figure 3B). Immunisation simulated at any time-point leads to lower GSK3β compared with no immunisation (black curve vs. coloured curves - Figure 3A) but there is no significant difference in active GSK3β pools by day 12 between the different interventions. However, according to the model, the reduction in active GSK3β at earlier time-points is not sufficient to significantly reduce levels of phospho-tau by day 12.

### Parameters/reactions which will affect model predictions

Plots of individual simulations show that the cell to cell variability in levels of Aβ, tau and the kinetics of their aggregation are fairly small (Figure S1). Therefore we used a deterministic model to carry out parameter scans in COPASI [44]. We first checked how the deterministic model compared with the stochastic model for the different interventions and found that there were some differences due to the low numbers of Aβ molecules (Figure S2). The best agreement between the models occurred for the intervention on day 4 and so we used this model for the parameter scans. We varied each parameter in turn from half to double its initial value and examined the effect on levels of Aβ, tau, activated micoglia and ROS over a 12 day time course. Out of 73 parameters, 22 had no effect, 11 had very small non-significant effects and 40 had signficant effects on one or more of the species examined. The latter parameters are directly involved either in the aggregation process or in the DNA damage response. The results are summarized in Table 1 and plots from a selection of the key parameters are shown in Figures S3 and S4. It can be seen that the parameters which are directly involved in the DNA damage response effect Aβ, tau, activated microglia and ROS simultaneoulsy and always in the same direction (i.e. all levels are increased or decreased in response to the increase in the parameter value). This means that the model predicts that an increase (or decrease) in DNA damage results in an increase (or decrease) in both Aβ and tau aggregation. On the other hand, changes in the values of parameters which only affect aggregation processes may affect either Aβ and tau aggregation (e.g. production of Aβ via GSK3β, Figure S4B) or only tau aggregation (e.g. tau binding to microtubules, Figure S3C). We found that there was no parameter which only affected Aβ without affecting tau aggregation. This suggests that reducing levels of Aβ may reduce the formation of phospho-tau and tangles in support of the amyloid hypothesis [45], [46].

**Table 1:** Summary of parameter scans showing the effect of increasing the parameter value by a factor of two on the levels of Aβ, tau, glia and ROS.

Parameter name	DNA damage response	Aggregation process	Soluble Aβ	Aβ plaques	Tau_P2	NFT	Activated glia	ROS
*k_actglia1_*		✓					↑	
*k_actglia2_*		✓					↑	
*k_aggTauP2_*		✓			↓	↑		
*k_binAbantiAb_*		✓	↓		↓	↓	↓	↓
*k_binMTTau_*		✓			↓	↓		
*k_degAbeta_*		✓	↓	↓	↓	↓	↓	↓
*k_degAntiAb_*		✓	↑	↑	↑	↑	↓	
*k_dephosTau_*		✓			↓	↓		
*k_dimerAbeta_*		✓	↓	↑	↓	↓	↑	↓
*k_disaggAbP1_*		✓	↓	↓	↓	↓	↓	↓
*k_genROSPlaque_*		✓	↑	↑	↑	↑	↑	↑
*k_inactglia1_*		✓					↓	
*k_inactglia2_*		✓					↓	
*k_pf_*		✓	↓	↓	↓	↓	↓	↓
*k_pg_*		✓	↑	↑	↑	↑	↑	↑
*k_pghalf_*		✓	↓	↓	↓	↓	↓	↓
*k_phosTauGsk3bp53_*		✓			↑	↑		
*k_prodAbeta2_*		✓	↑	↑	↑	↑	↑	↑
*k_relMTTau_*		✓			↑	↑		
*k_synTau_*		✓			↑	↑		
*k_actATM_*	✓		↑	↑	↑	↑	↑	↑
*k_binGsk3bp53_*	✓		↑	↑	↑	↑	↑	↑
*k_binMdm2p53_*	✓		↓	↓	↓	↓	↓	↓
*k_damROS_*	✓		↑	↑	↑	↑	↑	↑
*k_degMdm2mRNA_*	✓		↑	↑	↑	↑	↑	↑
*k_degp53mRNA_*	✓		↓	↓	↓	↓	↓	↓
*k_dephosMdm2_*	✓		↓	↓	↓	↓	↓	↓
*k_inactATM_*	✓		↓	↓	↓	↓	↓	↓
*k_Mdm2Pub_*	✓		↑	↑	↑	↑	↑	↑
*k_phosMdm2_*	✓		↑	↑	↑	↑	↑	↑
*k_relGsk3bp53_*	✓		↓	↓	↓	↓	↓	↓
*k_remROS_*	✓		↓	↓	↓	↓	↓	↓
*k_repair_*	✓		↓	↓	↓	↓	↓	↓
*k_synMdm2_*	✓		↓	↓	↓	↓	↓	↓
*k_synMdm2mRNA_*	✓		↓	↓	↓	↓	↓	↓
*k_synMdm2mRNAGsk3bp53_*	✓		↓	↓	↓	↓	↓	↓
*k_synp53_*	✓		↑	↑	↑	↑	↑	↑
*k_synp53mRNA_*	✓		↑	↑	↑	↑	↑	↑
*k_synp53mRNAAbeta_*	✓		↑	↑	↑	↑	↑	↑

Key: ↓ 2-fold decrease in parameter value; ↑2-fold increase in parameter value.

In order to gain more insight we ranked the top 40 parameters that control the change in the model output. We first ranked them according to the amount of reduction in the maximum size of plaques. To do this, we calculated the % change in maximum plaque size when the parameter value was halved or doubled compared to the default parameter value. Only 28 parameters affected plaque size. In 14 cases, increasing the parameter value reduced plaque size, whereas for the other 14 cases, decreasing the parameter value reduced plaque size. We ranked the parameters according to their effect on plaque size in Table 2 where it can be seen that the parameters involved in Aβ production, plaque growth and plaque disaggregation had the largest effect (see also Figure S5 for a diagram of the reactions corresponding to the top 28 parameters). Note that *k_binGSK3bp53_* and *k_relGSK3bp53_* affect Aβ production as we assumed that GSK3β bound to p53 increased production of Aβ. We also ranked the parameters according to their effect on decreasing levels of soluble Aβ, since this species may also be toxic. We found that each parameter affected plaques and soluble Aβ in the same direction which may be surprising as for example, we might expect that decreasing plaque size would lead to an increase in soluble Aβ. However, the soluble pool included monomers and dimers and on closer examination, we found that levels of dimers did increase when plaques decreased. However, a reduction in plaque size also reduced levels of ROS which then led to less Aβ production via activatedGSK3β. Although most of the parameters had similar ranks for plaques and soluble Aβ, (Pearson's product-moment correlation = 0.493, p-value = 0.0077, indicating a fairly strong correlation between the two sets of ranks), there were four notable exceptions. The parameters for plaque disaggregation and for plaque growth are ranked 2, 5 and 6 for their effect on plaque size but are ranked 23, 24 and 25 for their effect on soluble Aβ. The fourth parameter *k_dimer_* has only a small effect on plaques (ranked 28th) but decreasing this parameter reduces soluble Aβ by nearly 30% (ranked 4^th^). This is due to our assumption that monomers are degraded much more rapidly than dimers (which are only degraded when bound to antibodies). The parameters corresponding to the addition of antibodies did not affect plaque size in this analysis as we only looked at maximum plaque size which occurred before the addition of antibodies.

**Table 2:** Parameters ranked in order of their effect on Aβ Plaques.

Rank	Parameter name	Direction of parameter change	% decrease in maximum plaque size	Rank for soluble Aβ	Direction of parameter change	% decrease in maximum soluble Aβ levels
1	*k_prodAbeta2_*	↓	97.91	1	↓	45.81
2	*k_disaggAbP1_*	↑	96.77	23	↑	10.11
3	*k_binGSK3bp53_*	↓	96.30	2	↓	39.30
4	*k_relGSK3bp53_*	↑	96.29	3	↑	39.29
5	*k_pghalf_*	↑	93.43	24	↑	9.42
6	*k_pg_*	↓	57.24	25	↓	5.61
7	*k_synMdm2_*	↑	53.02	5	↑	24.96
8	*k_degMdm2mRNA_*	↓	51.81	6	↓	24.68
9	*k_binMdm2p53_*	↑	50.91	7	↑	23.96
10	*k_synp53_*	↓	50.46	8	↓	23.60
11	*k_degp53mRNA_*	↑	49.94	11	↑	23.49
12	*k_phosMdm2_*	↓	49.15	9	↓	23.58
13	*k_dephosMdm2_*	↑	49.09	10	↑	23.56
14	*k_Mdm2PUb_*	↓	48.29	12	↓	23.36
15	*k_synp53mRNA_*	↓	41.34	13	↓	19.59
16	*k_actATM_*	↓	39.60	14	↓	17.90
17	*k_damROS_*	↓	39.60	15	↓	17.90
18	*k_inactATM_*	↑	38.71	17	↑	17.64
19	*k_degAbeta_*	↑	36.96	16	↑	17.66
20	*k_remROS_*	↑	33.78	19	↑	16.06
21	*k_synMdm2mRNA_*	↑	33.09	18	↑	16.78
22	*k_genROSAbeta_*	↓	33.00	21	↓	11.76
23	*k_pf_*	↑	29.87	27	↑	2.54
24	*k_repair_*	↑	24.51	20	↑	12.96
25	*k_synMdm2mRNAGSK3bp53_*	↑	22.13	22	↑	11.44
26	*k_genROSPlaque_*	↓	5.03	26	↓	4.39
27	*k_synp53mRNAAbeta_*	↓	3.77	28	↓	2.32
28	*k_dimerAbeta_*	↓	3.10	4	↓	28.69

Key: ↓ 2-fold decrease in parameter value; ↑2-fold increase in parameter value.

The effect of parameters on tangle formation and phosphorylation of tau are shown in Table 3 and Figure S5. We found that 36 parameters had effects which included all those that had effects on Aβ and in addition 8 parameters that were specific to tau (*k_dephosTau_*, *k_phosTauGsk3bp53_*, *k_binMTTau_*_,_ *k_relMTTau_ k_aggTauP2_*, *k_binAbantiAb_*, *k_synTau_*, *k_degAntiAb_*). The two parameters involving antiAb affected tau and not Aβ as maximum levels of Aβ occurred before immunization, whereas our model predicted that tau continued to increase after this intervention. Increasing the effectiveness of the treatment had beneficial effects on tau due to the lowering in ROS (see Figures S3 and S4). It is interesting to note that none of the parameters that were specific to tau had any effects on Aβ level or its aggregation whereas the reverse was not true. This suggests that reactions involving tau are downstream of Aβ and do not feed back into the system. The parameters which had most effect on tangle formation were those that involved phosphorylation of tau by GSK3β. It may be surprising that the parameters affecting Mdm2 and p53 ranked so highly but this is because increasing the binding of Mdm2 to p53 prevents binding of GSK3β to p53 and lowers the activity of GSK3β. There was very little difference in the rankings for the parameters that affected tangle formation and phospho-tau with the top 13 being identical (Pearson's product-moment correlation = 0.957, p-value<2.2e-16, indicating a very strong correlation between the two sets of ranks). Although we assumed that free pools of unphosphorylated tau could aggregate, the parameter for aggregation of phosphorylated tau was set to be 10 times higher. Therefore phosphorylation of tau is closely linked to its aggregation. The other 4 parameters only affected activation of glia as the maximum plaque size occurred before glia activation. Increasing the activation rate of glia led to higher levels of activated glia and a slightly more rapid decline in plaques (data not shown). However, as plaques decline fairly rapid with the default parameters, the effect is not significant.

**Table 3:** Parameters ranked in order of their effect on tangles.

Rank	Parameter	Direction of parameter change	% decrease in maximum NFT level	Rank for effect on phospho-tau	Direction of parameter change	% decrease in maximum phospho-tau level
1	*k_binGSK3bp53_*	↓	94.40	1	↓	72.26
2	*k_relGSK3bp53_*	↑	94.40	2	↑	72.25
3	*k_dephosTau_*	↑	92.65	3	↑	70.60
4	*k_phosTauGsk3bp53_*	↓	92.13	4	↓	69.88
5	*k_synMdm2_*	↑	80.02	5	↑	54.67
6	*k_degMdm2mRNA_*	↓	79.89	6	↓	54.67
7	*k_binMdm2p53_*	↑	78.66	7	↑	53.33
8	*k_phosMdm2_*	↓	78.29	8	↓	53.20
9	*k_dephosMdm2_*	↑	78.26	9	↑	53.17
10	*k_Mdm2PUb_*	↓	78.16	10	↓	53.16
11	*k_synp53_*	↓	77.91	11	↓	52.49
12	*k_degp53mRNA_*	↑	77.84	12	↑	52.49
13	*k_synp53mRNA_*	↓	72.03	13	↓	46.72
14	*k_actATM_*	↓	69.75	16	↓	42.23
15	*k_damROS_*	↓	69.75	17	↓	42.23
16	*k_inactATM_*	↑	69.58	18	↑	41.82
17	*k_binMTTau_*	↑	68.72	14	↑	43.29
18	*k_remROS_*	↑	68.55	20	↑	38.84
19	*k_relMTTau_*	↓	68.45	15	↓	43.04
20	*k_repair_*	↑	66.33	22	↑	32.45
21	*k_synMdm2mRNA_*	↑	65.00	19	↑	41.02
22	*k_genROSAbeta_*	↓	63.49	24	↓	26.55
23	*k_prodAbeta2_*	↓	60.96	21	↓	33.24
24	*k_synMdm2mRNAGSK3bp53_*	↑	50.49	23	↑	29.01
25	*k_aggTauP2_*	↓	47.42	34	↓	3.63
26	*k_binAbantiAb_*	↑	47.42	35	↑	3.63
27	*k_synTau_*	↓	28.75	26	↓	11.05
28	*k_dimerAbeta_*	↑	25.41	31	↑	7.66
29	*k_degAbeta_*	↑	23.76	25	↑	15.83
30	*k_disaggAbP1_*	↑	12.31	28	↑	9.06
31	*k_synp53mRNAAbeta_*	↓	10.67	33	↓	6.07
32	*k_pghalf_*	↑	10.39	29	↑	8.92
33	*k_genROSPlaque_*	↓	8.13	27	↓	10.51
34	*k_pg_*	↓	7.55	30	↓	8.72
35	*k_degAntiAb_*	↓	7.41	-	↓	0.00
36	*k_pf_*	↑	6.10	32	↑	6.77

Key: ↓ 2-fold decrease in parameter value; ↑2-fold increase in parameter value.

## Discussion

We extended our previous model of GSK3 and p53 and their effects on the aggregation of Aβ and tau [33] to include reactions that describe a simulated immunisation againt Aβ. Our approach is novel as we considered in addition to Aβ aggregation, the role of tau and included other molecular mechanisms which may contribute to the aggregation process in the ageing brain, and the effects of Aβ intervention. The motivation for extending our previous model was the observation that immunotherapy can reduce tau phosphorylation. We wanted to examine whether our model would also predict this following a simulated immunisation against Aβ. Our modified model included assumptions about the effects of immunisation on Aβ but we did not include any direct mechanism for the effects on tau. The model includes indirect interactions between Aβ and tau via GSK3β but due to the complexity of the model, the effects on tau were not obvious prior to carrying out simulations. Our model predicted reduced tau phosphorylation after immunisation as observed giving support to our model assumptions. However, further experimental tests of the model predictions are required to establish its accuracy. If there is discrepancy, this will lead to refinement of hypotheses and modification of the model. This actual process can lead to further understanding to the underlying mechanisms. Other mechanisms which may be important and are not currently included in our model are discussed below.

We chose to mimic the effect of both active immunisation with full length AB42 (AN1792) and passive immunisation with an antibody that works by enhancing Aβ degradation, plaque disaggregation and phagocytosis of plaques via activated microglia. There are other newer antibodies such as Solanuzumab which have different modes of action and so the model would need to be modified to test these. However, modifications of SBML models are straight forward to carry out and this could be done in the future. We included the effects of antibodies on soluble Aβ and plaques, the activation of microglia, and the effects of activated microglia on plaques and levels of ROS. Our model predicted that immunisation reduces levels of plaques but that pools of soluble Aβ were only reduced by small amounts. The activity of GSK3β and levels of phospho-tau were both reduced immediately after immunisation and then remained at fairly constant levels in accordance with experimental data. This was due to the reduction in ROS levels after clearance of plaques. We used our model to study the effect of passive and active immunotherapy of established disease, and the use of immunotherapy as a preventive measure for Alzheimer's disease.

We chose to base our model on a cellular system and used a time period of 12 days for the simulations. The main reason for this choice is that is not currently feasible to run complex models over long time periods using stochastic simulation since each individual simulation would take many hours to complete and many repeat simulations are required. Experimental cellular models induce Aβ aggregation by either the addition of Aβ to the extracellular fluid [38] or by the addition of rotenone [47]. In both systems the kinetics of Aβ aggregation is similar, with aggregation being observed by 24 h and maximal aggregation by 2 days. The limitation of using a cellular model, (either experimentally or in a computer model) is that it may not be valid to extrapolate findings to human ageing. However, cellular models do provide valuable information on the potential underlying molecular mechanisms in the aggregation process and can aid understanding of how interventions such as immunisation can ameliorate the process. It would be possible to adapt the current model to run simulations over longer timescales to mimic human ageing if we used deterministic simulation and modified the parameters involved in the aggregation process.

The model contains many parameters and so we carried out parameter scans to examine which oness affect the levels of soluble Aβ, plaques, phospho tau, tangles, activated glia and ROS.

**Figure 4:**
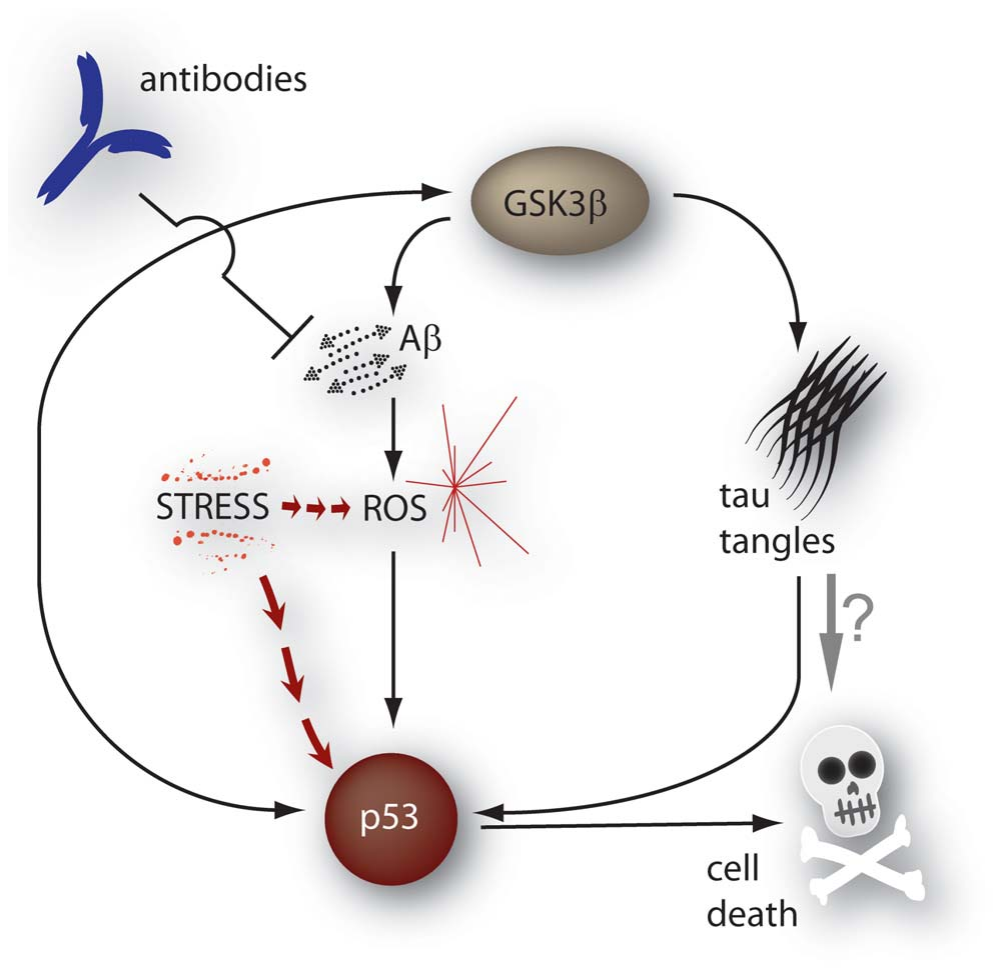
Cycle of reactions leading to aggregation of Aβ and tau. Immunisation reduces aggregation of both Aβ and tau by breaking the cycle. However stress within the cell may still cause activation of p53 and GSK3β so that the aggregation process of tau continues.

The parameters which are involved in DNA damage and the DNA damage response (e.g. *k_damROS_*, *k_actATM_*, *k_repair_*) had simultaneous affects on ROS levels, soluble Aβ, plaques, phospho-tau and tangles. This is due to the cycle of events and the self-amplifying loop of an increase in GSK3β activity, Aβ levels, ROS, p53 activation, further increased activity of GSK3 and hyperphosphorylation of tau (Figure 4) and a detailed figure showing the key reactions and parameters is included in the supplementary information (Figure S5). This cycle has previously been described in detail and showed how both familial and sporadic AD can be explained by a unified hypothesis due to the fact that the cycle can start at any point [48]. Breaking the cycle by immunisation will reduce plaques but without altering other stress within the cell which may lead to continued activation of p53 and GSK3β and thus to hyperphosphorylation of tau and accumulation of tangles. Although the exact neurotoxic species of tau has yet to be conclusively identified [49], there is a general consensus that abnormal and hyperphosphorylated tau has detrimental effects, leading to loss of neurons, strongly suggesting that therapies which just target Aβ may not be beneficial. Additional interventions which reduce cellular stress and/or reduce the activity of GSK3β are likely to be required in order to slow down disease progression. Our data with decrease of phospho tau without any changes in tangles [10], [17] and the absence of clinical improvement [11] support this hypothesis.

We chose to focus on GSK3β and p53 due to the finding that both proteins are upregulated in AD and the relevance of GSK3β in AD [50], [51]. There are likely to be many other pathways and proteins involved, but we still lack knowledge of what is actually important in the disease process. Other kinases are also involved in tau phosphorylation, such as CDK5, and the effects of phosphorylation on the affinity of tau for microtubules is much more complex than we have modelled here [52]. However, there is still a lot to learn about the details and it will be possible to modify our model as new data emerge. Our model was originally developed to test hypotheses about the assumed pathways [20] and we believe that further experiments to examine the role of GSK3β and p53 in AD would increase our understanding of the processes involved. We do not claim to have produced a model that encompasses all aspects of the disease process and as for all simplified models, great care should be taken when extrapolating data to the clinic. However, it will be possible to extend the current model to include additional pathways, as additional experimental and clinical data emerge, to provide a more complete description. Another aspect of the model that could be extended is the aggregation process. We only included one pathway for Aβ aggregation and assumed that the building blocks of plaques are dimers [53] as we wanted to keep the model as simple as possible, and there is controversy over the actual mechanisms of aggregation in the brain. For example, it has been suggested that low molecular mass oligomers extracted from AD brain may be artefacts induced by the use of detergents in the extraction process [54]. However, even if dimers are not detected in the human brain, this does not preclude the possibility that they are present at a level too low to detect but are important in the aggregation process. As our model predicts that dimers are very unstable and either dissociate into monomers or rapidly aggregate. This means that levels of dimers may always be too low to be detected. If desired, modelling could be used to test different hypotheses of the aggregation process by building a set of models with different schemes for the aggregation mechanism and then comparing the model predictions.

An interesting extension of the model would be to include the connection between Aβ and synaptic activity. A recent study suggests that synaptic activity leads to an increase in Aβ production via endocytosis, and that Aβ inhibits synaptic activity [55]. We hypothesise that including this negative feedback loop in the model would keep both Aβ levels and synaptic activity within the normal range. It has been suggested that low levels of Aβ may have this functional role (reviewed in [56]), however, the negative feedback loop may be disrupted in AD. For example, an increase in Aβ due to other pathways may lead to formation of Aβ oligomers and/or plaques which may interfere with synaptic activity. When more information becomes available on the link between Aβ and synaptic activity, it will be possible to examine the effect of disruption of the feedback loop on Aβ secretion and aggregation.

The disparity between the promising outcomes seen in preclinical transgenic animal models and the disappointing results of early phase clinical trials has necessitated additional reflection on mode of action of Aβ immunotherapy and the best strategies for future clinical trials. Based on cognitive and pathological measures it has been widely acknowledged that the best mouse models fall short of fully recapitulating Alzheimer's disease, but this conclusion is made inescapable by the outcomes of the immunotherapy trials carried out to date.

It could be argued that the current “gold standard” for a mouse model would be the triple transgenic expressing clinically relevant mutant isoforms of presenilin (M146V), amyloid precursor protein (the Swedish mutation), and tau (P301L) [57]. Passive immunisation with Aβ-specific antibodies provided clear benefit to triple transgenic mice at the histological level [58]. Taken in isolation this finding would not provide justification for a clinical trial (in this study the antibody was delivered by direct injection into the hippocampus, obviating issues relating to the blood brain barrier) but is notable in the current context for its mechanistic insights. Though the immunotherapy was directed at Aβ there was a reduction in tau pathology at early stages (the extensively phosphorylated tau of later stages could not be cleared). These data suggest that the passive Aβ immunisation would be efficacious before the formation of tau tangles, in agreement with our simulations.

One unavoidable limitation of the best available transgenic mouse models is that they incorporate mutations associated with the inherited forms of AD, and may inadequately mimic the more numerous sporadic forms of the disease (reviewed in [59]). Early (presymptomatic) intervention in the form of active or passive immunisation may be plausible in individuals deemed to be at high risk of AD due to family history and/or genetic testing, but with the currently associated risks and uncertain benefits, prophylactic Aβ immunisation is inconceivable. The situation may change as models become available for sporadic AD. A recent report of a “natural” model (age-related changes strongly reminiscent of AD in the rodent *Octodon degus*) may be grounds for optimism in this regard [60].

The great advantage of the computational modelling approach is the ease with which parameters can be updated and models adapted as new information becomes available. In its current form, our model is sensitive to changes in some (but not all) parameters, and some of these parameters represent druggable targets for which clinical and experimental data may be forthcoming. As an example, it has already been demonstrated that inhibition of GSK3β is neuroprotective in an AD mouse model [61]. On the other hand, adverse effects of GSK3β inhibition have also been reported to have neurotoxic effects in mice models due to induction of apoptosis by nuclear factor of activated T-cells (NFAT) [62]. Although it will be necessary to extend our current model to test these effects, computer modelling could be a powerful tool for examining the possible effects of potential targets. There is also *in vivo* evidence for the protective effects of antioxidants, including the natural plant phenol resveratrol [63]. It would be relatively straightforward to adapt the computer model to simulate such interventions, to predict possible outcomes were such agents used together or in combination with immunisation strategies, and to incorporate findings from relevant clinical trials as their outcomes are published.

## Conclusions

This paper describes how our mathematical model can simulate and predict the effects of Aβ immunisation in Alzheimer's disease. We believe that the model will become increasingly accurate as new mechanistic details of the relevant pathways become available, and suggest that the mathematical model will be useful in testing possible interventions prior to clinical trials.

## Methods

### Model construction

The model of Proctor & Gray [20] was modified to include processes involved in Aβ immunisation. Before describing how the model was modified, we give a brief description of this model which we refer to as the GSK3 model. The GSK3 model was constructed to investigate the relationship between GSK3β, p53, Aβ and tau. It was built in a modular way and includes components for DNA damage, p53 regulation, GSK3 activity, Aβ turnover, tau dynamics and the aggregation of Aβ and tau. In the module for p53 regulation we assumed that p53 binds to the E3 ligase Mdm2 and is then ubiquitinated and targeted for degradation by the 26S proteasome [64]. Under normal (unstressed) conditions, both p53 and Mdm2 are kept at low basal levels. The module for the DNA damage response includes detail of p53 activation which occurs after DNA damage due to p53 phosphorylation which prevents p53 binding to Mdm2 and so it is no longer degraded. When p53 levels are elevated it can bind to GSK3β which increases the activity of both proteins [43], [65]. In the tau module, we assumed that tau is continuously being phosphorylated (by GSK3β) [66] and dephosphorylated (by PP2) [67]] to regulate its binding to microtubules. When GSK3β activity is increased, more tau is phosphorylated and tau may then start to aggregate. In the Aβ turnover module we assumed that Aβ is continually produced and degraded but when GSK3β and p53 bind, the production of Aβ is increased which in return increases the production of p53. Although the precise mechanism of increased Aβ production via GSK3 is still not clear, studies have shown that GSK3 inhibitors reduce APP processing (reviewed in [68]). We also assumed that soluble Aβ leads to an increase reactive oxygen species (ROS) [22] which may then lead to further DNA damage and increased activation of p53. This vicious cycle of events has been described in a recent publication [48]. The full details of the GSK3 model are available in an open access journal [20] and the SBML code is available from the Biomodels database (BioModels ID:BIOMD0000000286) [69]. The additional species and reactions for the modified model are shown in Tables 4, 5, and 6 and the assumptions made are detailed below.

**Table 4:** Additional species for the immunotherapy model. All other species in the model are given in Proctor & Gray [20].

Species id	Description	Initial value
GliaI	Inactive glia	100
GliaP1, GliaP2	Glia associated with plaques but not able to phagocytose	0
GliaA	Active glia which can phagocytose	0
antiAb	Antibody against amyloid-beta	0
AbP_GliaA	Abeta plaque bound to active glia	0
Abeta_antiAb	Abeta monomer bound to antibody	0
AbDim_antiAb	Abeta dimer bound to antibody	0
AbDim	Abeta dimer (replaces species AggAbeta)	0
AbP	Abeta plaques (replaces species AbetaPlaque)	0

**Table 5:** Additional reactions for the immunotherapy model. All other reactions in the model are given in Proctor & Gray [20].

Reaction id	Reactants	Products	Kinetic rate law
Glia ActivationStep1	GliaI, AbP	GliaP1, AbP	k_actglia1_*GliaI*AbP
Glia ActivationStep2	GliaP1, AbP	GliaP2, AbP	k_actglia1_*GliaP1*AbP
Glia ActivationStep2	GliaP2, antiAb	GliaA, antiAb	k_actglia2_*GliaP2*antiAb
GliaInactivationStep1	GliaA	GliaP2	k_inactglia1_*GliaA
GliaInactivationStep2	GliaP2	GliaP1	k_inactglia2_*GliaP2
GliaInactivationStep3	GliaP1	GliaI	k_inactglia2_*GliaP1
AbetaPlaqueBindingToGlia	GliaA, AbP	AbP_GliaA	k_binAbetaGlia_*GliaA*AbP
AbetaPlaqueReleaseFromGlia	AbP_GliaA	GliaA, AbP	k_relAbetaGlia_*AbP_GliaA
PlaqueClearanceByGlia	AbP_GliaA	GliaA	k_degAbetaGlia_*AbP_GliaA
ROSgenerationByGlia	AbP_GliaA	AbP_GliaA, ROS	k_genROSGlia_*AbP_GliaA
ROSgenerationByPlaques	AbP	AbP, ROS	*k_genROSplaque_* * AbP
AntiAbRemoval	antiAb	Sink	k_degAntiAb_*antiAb
AntiAbBindingToAbeta	Abeta,antiAb	Abeta_antiAb	k_binAbantiAb_*Abeta*antiAb
AntiAbBindingToAbetaDimers	AbDim,antiAb	AbDim_antiAb	k_binAbantiAb_*AbDim*antiAb
AbetaDegradationViaAntiAb	Abeta_antiAb	antiAb	k_degAbetaAntiAb_*Abeta_antiAb
AbetaDimerDegradViaAntiAb	AbDim_antiAb	antiAb	k_degAbetaAntiAb_*AbDim_antiAb
PlaqueDisaggregation	AbP	Abeta	k_disaggAbeta1_*AbP
PlaqueDisaggregationViaAntiAb	AbP, antiAb	Abeta,antiAb	k_disaggAbeta2_*AbP*antiAb
AbetaDedimerisation	AbDim	Abeta	*k_dedimer_* * AbDim

**Table 6:** Values for additional parameters in the immunotherapy model. All other parameters in the model are given in Proctor & Gray [20].

Parameter	Value	Comment
*k_actglia1_*	6.0e-7 molecule^−1^s^−1^	Partial activation of microglia increases linearly with plaque levels.
*k_actglia2_*	6.0e-7 molecule^−1^s^−1^	Full activation of microglia takes about 1–2 days after addition of antibodies
*k_binAbantiAb_*	1.0e-6 molecule^−1^s^−1^	Soluble Aβ binds to antibodies within a few hours after their addition.
*k_binAbPGlia_*	1.0e-5 molecule^−1^s^−1^	Fully activated pool of microglia bind to plaques within 15–30 minutes
*k_dedimerAbeta_*	1.0e-6 s^−1^	Rate of disassociation of dimers to monomers is assumed to be low so that aggregation will start within one to two days.
*k_degAbetaAntiAb_*	1.5e-4 s^−1^	We assumed that antibodies increase Aβ degradation rate by an order of magnitude.
*k_degAbPGlia_*	5.0e-3 s^−1^	Plaques associated with fully activated microglia are rapidly degraded via phagocytosis.
*k_degAntiAb_*	2.75e-6 s^−1^	Half-life of antibodies ≈3 days.
*k_disaggAbP1_*	2.0e-4 s^−1^	Rate chosen to give a maximum plaque size of about 80.
*k_disaggAbP2_*	1.0e-6 molecule^−1^s^−1^	Antibodies disaggregate plaques at lower rate than normal.
*k_genROSGlia_*	2.0e-5 s^−1^	Microglia generate ROS at slightly lower rate as Aβ.
*k_genROSPlaque_*	2.0e-5 s^−1^	Plaques generate ROS at same rate as glia.
*k_inactglia1_*	5.0e-6 s^−1^	Inactivation occurs with a half-life of about 4 days.
*k_inactglia2_*	5.0e-6 s^−1^	Inactivation occurs with a half-life of about 4 days.
*k_relAbPGlia_*	5.0e-5 s^−1^	The release of Aβ from microglia without phagocytosis has lower probability than degradation of Aβ via activated microglia.

### Modelling the immunisation process

The exact mechanism by which Aβ immunisation removes Aβ is not clear but it is likely to be several different pathways. Since existing plaques are reduced after immunisation, it is assumed that antibodies result in disaggregation of plaques with microglia phagocytosing Aβ. A study involving APP transgenic mice suggests that there is a two phase mechanism of Aβ clearance after administration of antibodies [70]. The first phase occurs 4–24 hours after immunisation and is independent of microglial activation and results in clearance of diffuse Aβ deposits. The second phase, which occurs between 1 and 3 days, involves clearance of amyloid plaques in association with activation of microglia.

We modified our previous model by adding a species named “antiAb” to represent the addition of antibodies (i.e. passive immunisation) and another species named “Glia” to represent microglia.

To model the addition of antibodies at different time-points, we assumed that initially antiAb = 0 and used an event structure in the SBML code so that when the simulation time is equal to the chosen time of the intervention, antiAb is set to 50. We also modelled active immunisation and in this case, included a reaction for antiAb production to ensure that antibodies were continually produced.

Note that for simplicity we have ignored spatial aspects in this model at present. The following assumptions were made concerning the role of glia and antibodies:

- Glia can be in four states: inactive (GliaI), partially active glia (GliaP1, and GliaP2) which are attracted to plaques but do not engulf them [71] and active (GliaA) which are activated by immunotherapy [72]. We included two pools of partially activated glia in order to provide a longer time lag between partial and full activation. Initially all glia are inactive (i.e. not able to phagocytose Aβ significantly).
- The presence of plaques is required for partial activation of glia [71].
- The presence of antibodies is required for full activation of glia [72].
- Active or partially active glia can be inactivated [73].
- Fully active glia can bind to plaques and degrade them by phagocytosis [70].
- Active glia bound to plaques can generate ROS [74].
- Antibodies can disaggregate plaques [75].
- Antibodies enhance the degradation of soluble Aβ [76].
- Antibodies can be degraded (to mimic diffusion from cell).

The role of glia is still poorly understood and there is controversy over whether they are neuroprotective or neurotoxic [77]. From our assumptions we have included both effects of glia. On the one hand they are able to degrade plaques by phagocytosis but on the other hand, they increase the ROS production when bound to plaques.

### Kinetics of aggregation

We assume that the aggregation process starts with the formation of Aβ dimers from two monomers but that this reaction is reversible. We represent monomers and dimers by the species Abeta and AbDim, respectively in this model. In our previous GSK3-p53 model, we used the name AggAbeta to represent Aβ dimers but changed this terminology to be consistent with a recent model of Aβ aggregation [41]. Similarly we renamed the reactions and parameters. There is controversy over the role of dimers and small oligomers in the aggregation process since there is limited direct evidence of their existence in AD. Although they are extracted from AD brain, they may represent artefacts induced by the peptide's interaction with detergent [54]. Interestingly, our model predicts that levels of dimers are always very low (close to zero) . This is due to the assumptions that either dimers dissociate into monomers or rapidly initiate the aggregation process.

Many different schemes of the actual aggregation process from monomers to plaques have been proposed and modelled. The formation of a new plaque has been shown to be nucleation dependent leading to a lag phase. In an *in vitro* system as modelled here, the lag phase is relatively short (about 1 day) whereas in a human brain this would be on timescales many years. Once a plaque has formed, further growth is governed by first-order kinetics [78]. Different deposition mechansims have been proposed including monomers, dimers or preassemble oligomers [79]. In order to keep the model as simple as possible, we only included one pathway, and based on fairly recent experimental data, we assumed that dimers are the building blocks of aggregates [53]. However, our model could be adapted to add other pathways if desired.

Our previous model assumed that plaque formation was irreversible and so once the aggregation process started, the model predicted that plaques form rapidly and continue to grow. However, studies show that although plaques do form quickly, they soon reach a maximum size where no further growth is observed [80], [81]. Since soluble Aβ is continually produced, this observation suggests that plaque growth is not irreversible but that disaggregation takes place. This means that plaques are dynamic structures in which Aβ is being continually deposited and removed and when these two processes are in balance, the plaque appears to be stable. Previous modelling suggests that plaques are porous and so the disaggregation rate depends on the size of the plaque [82]. Therefore we use mass action kinetics to model plaque disaggregation, which means that the rate is proportional to the size. The rate of plaque growth depends on the level of soluble Aβ and it may be considered that it also depends on plaque size, since we might assume that large plaques have a greater chance of sequestering more Aβ than small plaques. However with this assumption the rate of growth would always be greater than the rate of disaggregation (unless the production of Aβ ceased). Therefore we assume that plaque growth initially increases linearly with plaque size but then reaches a maximum rate. We use a Hill function to model this as in our previous model of Aβ aggregation (see Figure 10 of [41]). The chosen parameters for plaque growth and plaque disaggregation resulted in a maximum plaque size of 75. In reality, plaques are much larger but it was necessary to use relatively low numbers for all the molecules in our model, so that we could carry out simulations within reasonable time-frames. We also speeded up the aggregation process to mimic an experimental cellular system. If we modelled the process over much longer time periods to represent the ageing process, then the aggregation process would be slower and larger plaques would form. Since we examined all the parameters to see which ones had most effect on plaque size, it would be straight-forward to modify parameter values in order to obtain larger plaques if desired (see Results section and Table 2). We also included a reaction for ROS generation via plaques, in addition to our previous modelled reactions for ROS generation via soluble Aβ, since recent data shows that ROS can be produced by Aβ in all aggregation states [21].

### Aβ turnover

We assume that Aβ is continually produced and degraded and that normally Aβ levels are kept at low basal levels. We also assume that if p53 activity is increased via its interaction with GSK3β, then production of Aβ is increased [83]. However, more importantly, it has been observed that Aβ degradation is impaired in AD [39]. We previously modelled turnover and aggregation kinetics of Aβ and found that we needed to assume that the rate of Aβ degradation declines with age in persons which develop AD [41]. This is a reasonable assumption, since it has also been shown that neprilysin, a major Aβ-degrading enzyme declines with age [84], [85]. Therefore we also assume a lower rate of Aβ degradation in AD.

The maximum size of plaque is mainly determined by the amount of soluble Aβ and so parameters which have greatest affect on plaque size include Aβ production and clearance rates. We used the rates from an experimental study which looked at levels of Aβ in the cerebrospinal fluid (CSF) [39]. They found that the rate of Aβ production (1.86e^−5^ molecules s^−1^) was the same in AD subjects and age-matched normal controls but that the degradation rate was lower in AD patients (1.5e^−5^ s^−1^ in AD compared to 2.1^−5^ s^−1^ for controls). It should be noted that both the production and degradation rate of Aβ is likely to be higher in neurons than in the CSF but it is the ratio of production/degradation which is more important in determining levels of soluble Aβ rather than the individual rates. We assumed that this ratio is the same in neurons as the CSF and so do not believe that even if we had data for actual rates in neurons that it would not make any qualitative differences to the model predictions. Therefore we used the AD degradation rate from this study and then adjusted the parameters for aggregation so that plaques start to form by about 2 days and reach a maximum size by about day 10. This was to allow the model to be simulated in a reasonable amount of time. However, this meant that plaques still formed even with the observed mean degradation rate in the normal controls. We found that we needed to set the normal degradation rate an order of magnitude higher than the AD rate. We previously built an individual module of Aβ turnover and aggregation in which we used the rates from the data for both normal and AD cases as we were able to run this model over a time scale of 100 years. However, in this model, we needed to assume that the degradation rate declines with age in the AD patients, (a more reasonable assumption than a constant lower degradation rate since birth). In the current model, in which we speed up the aggregation process over a time scale of days, we assume a constant degradation rate for AD, since ageing effects are not modelled here.

### Parameter scans

We carried out all the parameter scans using COPASI [86]and anaylsed the results in the R statistical package. Note that all parameters scans were carried out with slightly different values to three of the parameters (*k_genROSAbeta_* = 2e-5, *k_genROSGlia_* = 1e-5, *k_genROSPlaque_* = 1e-5) which resulted in slightly lower levels of AbP, NFT, Abeta,Tau_P2, Glia_A and ROS than those shown in Figures S3 and S4. However, we made our comparisons to the maximum values of the species with these default parameters so that the ranking and percentage changes are not affected.

### Details of model code and simulation

The model was encoded in the Systems Biology Markup Language(SBML) [36], a computer-readable format for network models using the SBML shorthand tool [87]. SBML is a modelling standard that allows models to be easily modified or extended. The code is publicly available from the Biomodels database (Biomodels ID: MODEL1212030000) [69] and is available as a supplementary file (Code S1). Simulations were carried out using the Gillespie algorithm [88] which is a method for doing exact stochastic simulation. Simulation data was plotted in R using the ggplot2 package [89].

## Supporting Information

Figure S1 **Individual plots for model with low Aβ degradation rate and no immunisation.** Six runs from 100 simulations are plotted. Key: orange = soluble Aβ; blue = Aβ plaques; green = phospho-tau; black = tau tangles; red = activated glia.
(TIF)

Figure S2 **Model predictions for levels of Aβ, tau, activated glia under different simulated interventions using a deterministic model.** A–D Simulated passive immunisation administered at different time-points: A Day 0; B Day 4; C Day 8; D Repeated immunisation at Day 0 and Day 7. E Simulated active immunisation. F No immunisation. Key: orange = soluble Aβ; blue = Aβ plaques; green = phospho-tau; black = tau tangles; red = activated glia.
(TIF)

Figure S3 **Varying model parameters, part 1.** Effect of varying a selection of model parameters from half to double its initial value. The thickness of the line is scaled to the parameter value with thicker lines representing higher values. A *k_actglia2_*; B *k_binAbetaGlia_*; C *k_binMTTau_*; D *k_degAbeta_*; E *k_degAntiAb_*; F *k_dephosTau_*; G *k_genROSPlaque_*; H *k_pf_*. Key: orange = soluble Aβ; blue = Aβ plaques; green = phospho-tau; black = tau tangles; red = activated glia, purple = ROS.
(TIF)

Figure S4 **Varying model parameters, part 2.** Effect of varying a selection of model parameters from half to double its initial value. The thickness of the line is scaled to the parameter value with thicker lines representing higher values. A *k_pg_*; B *k_prodAbeta2_*; C *k_actATM_*; D *k_binGsk3bp53_*; E *k_binMdm2p53_*; F *k_damROS_*; G *k_remROS_*; H *k_repair_*; . Key: orange = soluble Aβ; blue = Aβ plaques; green = phospho-tau; black = tau tangles; red = activated glia, purple = ROS.
(TIF)

Figure S5 **Key components of the model network.** Network diagram of the key components in the model network showing the reactions involving the most sensitive parameters. The labels on the reaction arrows starting with ‘P’ or ‘T’ indicate the rank of the parameter with respect to its effect on the maximum level of plaques and tangles respectively.
(TIF)

Code S1 **SBML code.** This file contains the SBML code for the model in which passive immunisation was mimicked at day 4 after the start of the simulation.
(XML)
