# Supplementary figures and images for: Investigating Interventions in Alzheimer's Disease with Computer Simulation Models
Source: PLoS One. 2013 Sep 16;8(9):e73631. doi: 10.1371/journal.pone.0073631 (PMC3782376; doi:10.1371/journal.pone.0073631)

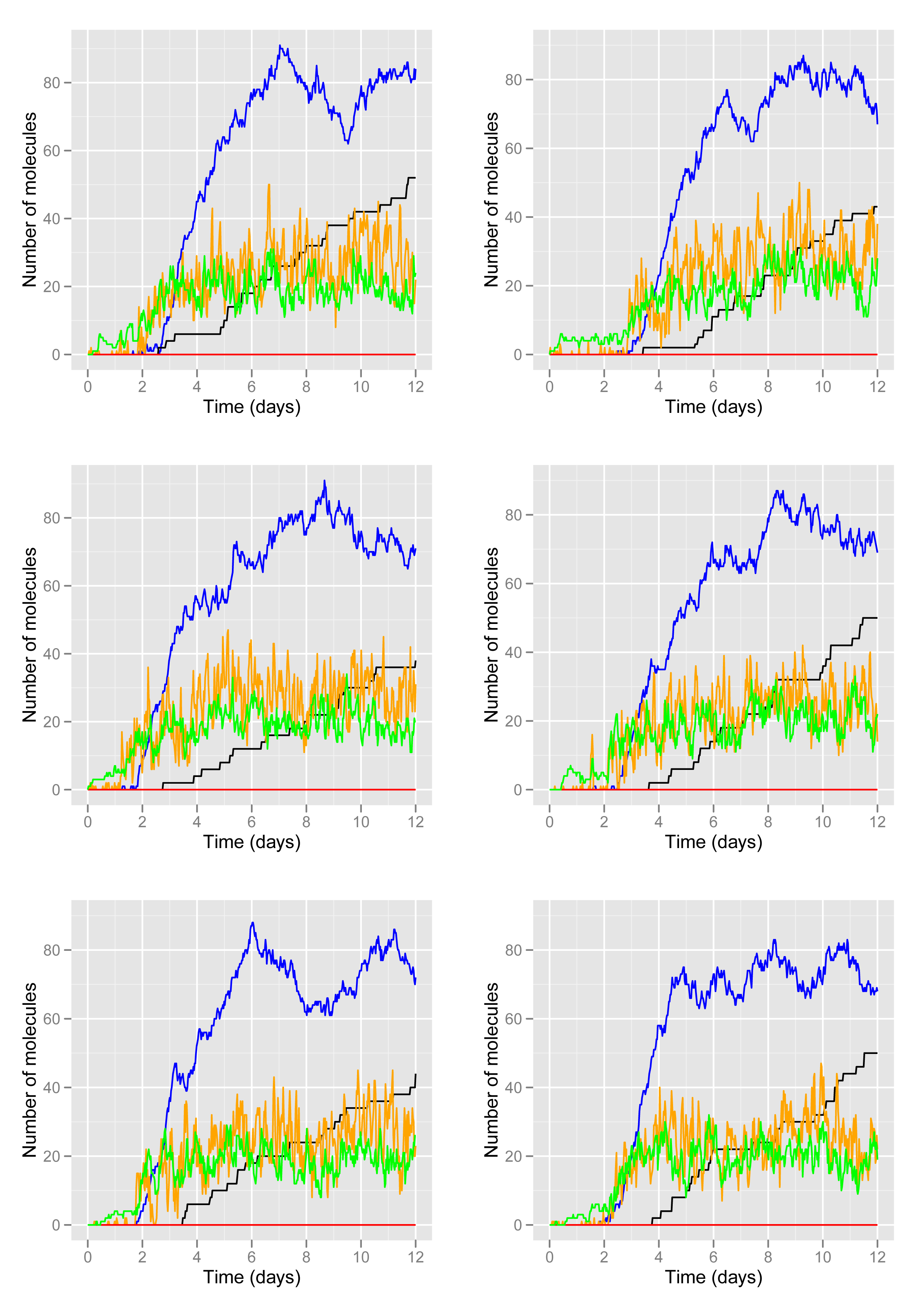

Supplement: Figure S1 — Individual plots for model with low Aβ degradation rate and no immunisation. Six runs from 100 simulations are plotted. Key: orange = soluble Aβ; blue = Aβ plaques; green = phospho-tau; black = tau tangles; red = activated glia. (TIF) [file pone.0073631.s001.tif]

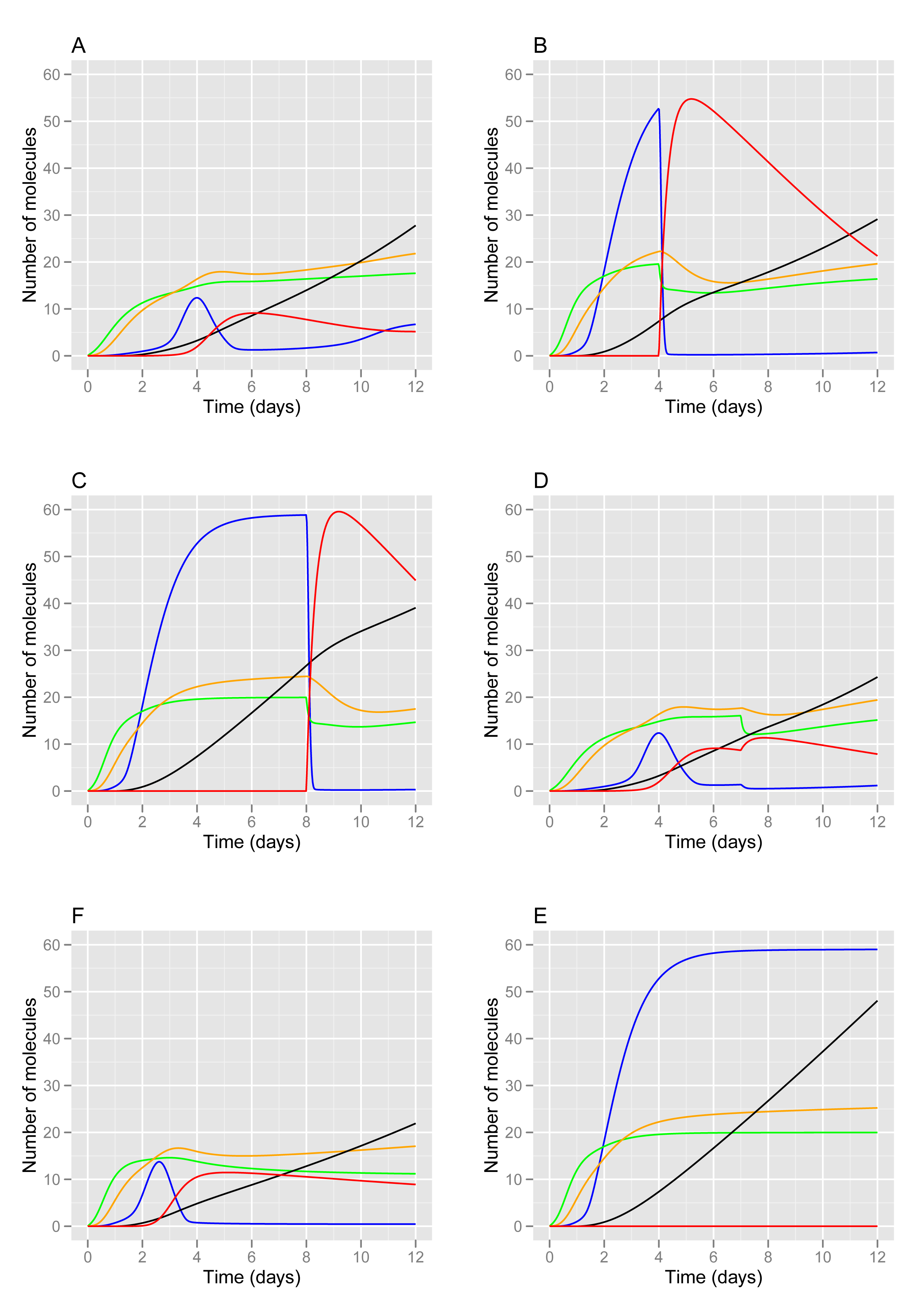

Supplement: Figure S2 — Model predictions for levels of Aβ, tau, activated glia under different simulated interventions using a deterministic model. A–D Simulated passive immunisation administered at different time-points: A Day 0; B Day 4; C Day 8; D Repeated immunisation at Day 0 and Day 7. E Simulated active immunisation. F No immunisation. Key: orange = soluble Aβ; blue = Aβ plaques; green = phospho-tau; black = tau tangles; red = activated glia. (TIF) [file pone.0073631.s002.tif]

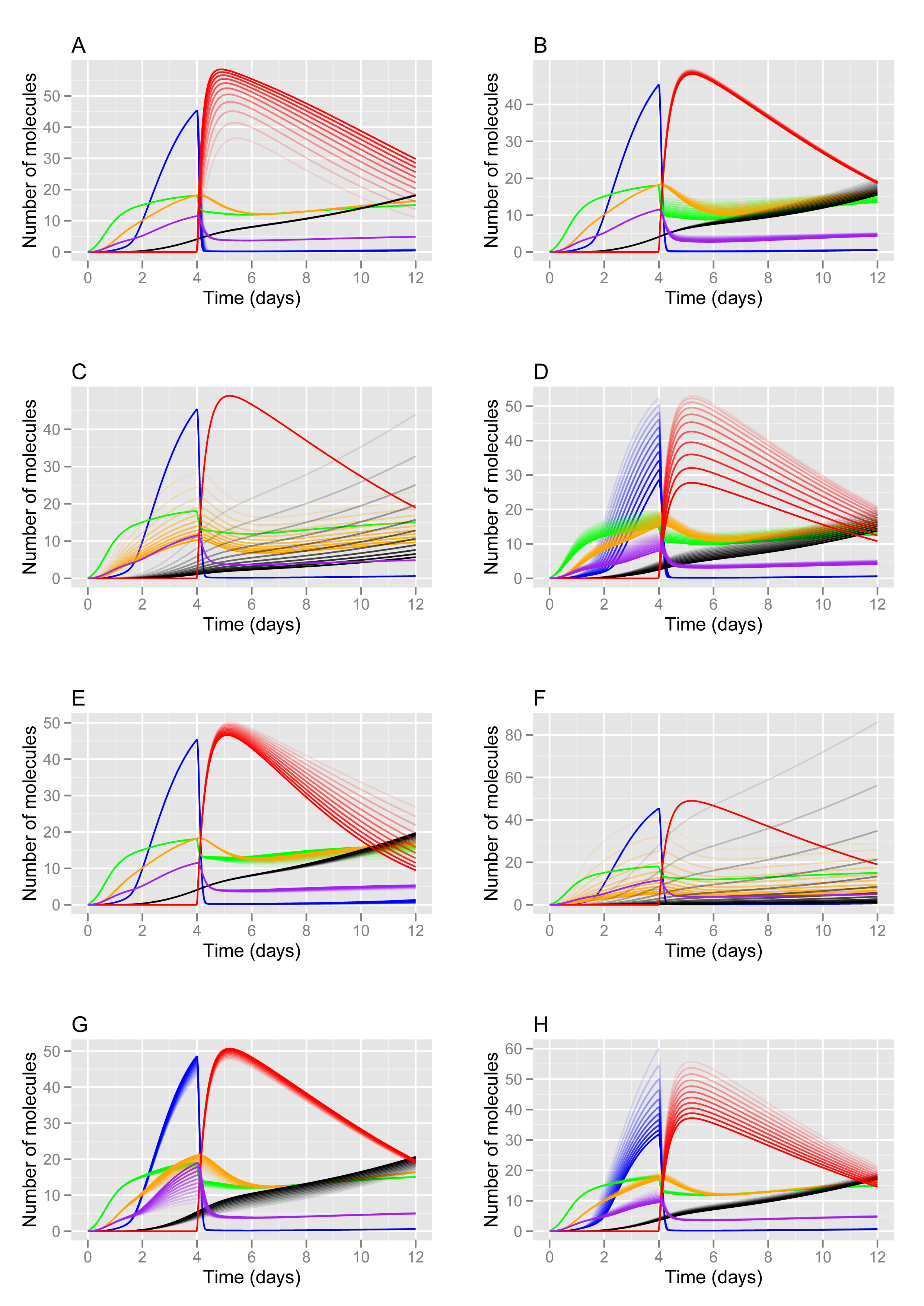

Supplement: Figure S3 — Varying model parameters, part 1. Effect of varying a selection of model parameters from half to double its initial value. The thickness of the line is scaled to the parameter value with thicker lines representing higher values. A kactglia2; B kbinAbetaGlia; C kbinMTTau; D kdegAbeta; E kdegAntiAb; F kdephosTau; G kgenROSPlaque; H kpf. Key: orange = soluble Aβ; blue = Aβ plaques; green = phospho-tau; black = tau tangles; red = activated glia, purple = ROS. (TIF) [file pone.0073631.s003.tif]

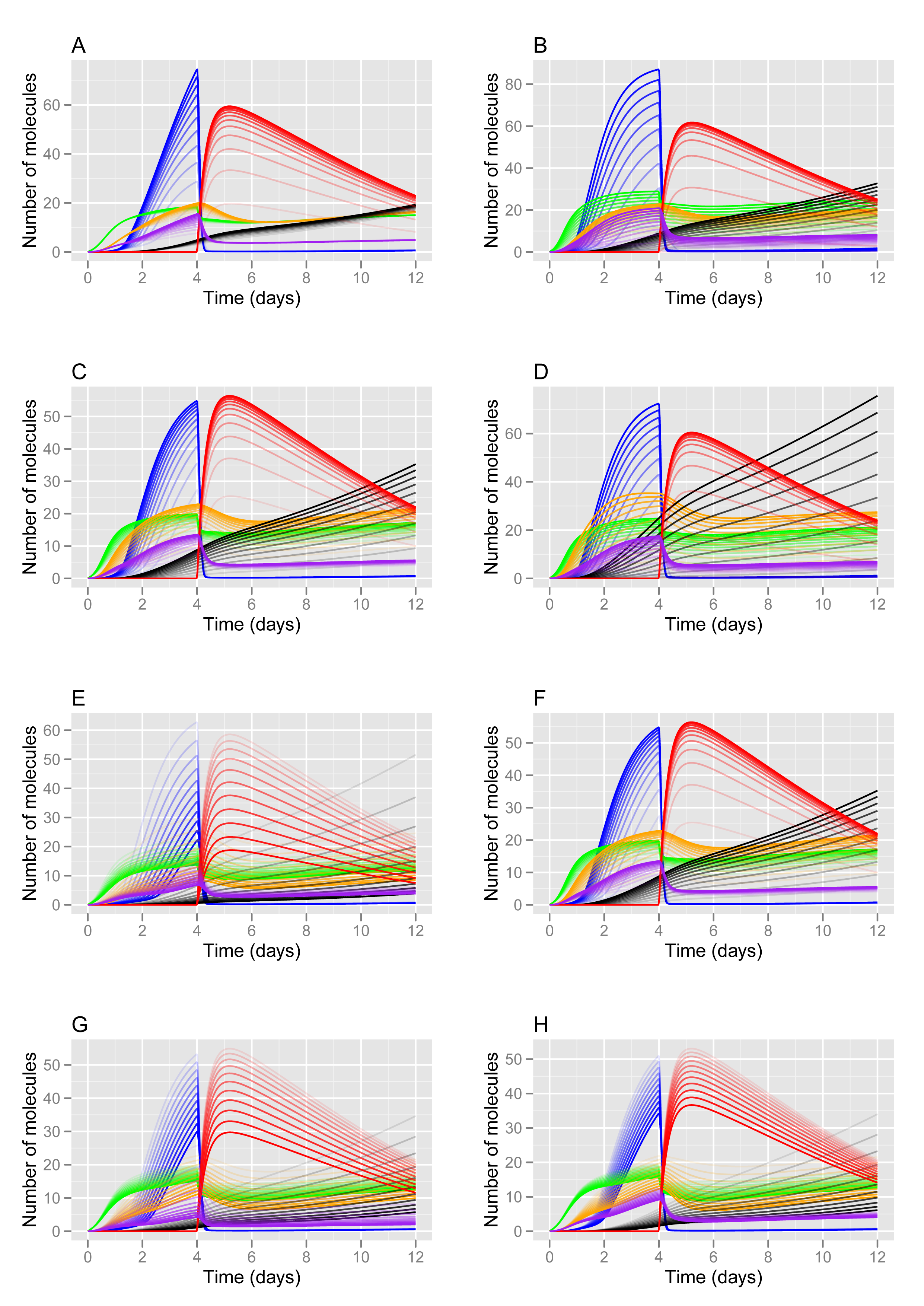

Supplement: Figure S4 — Varying model parameters, part 2. Effect of varying a selection of model parameters from half to double its initial value. The thickness of the line is scaled to the parameter value with thicker lines representing higher values. A kpg; B kprodAbeta2; C kactATM; D kbinGsk3bp53; E kbinMdm2p53; F kdamROS; G kremROS; H krepair; . Key: orange = soluble Aβ; blue = Aβ plaques; green = phospho-tau; black = tau tangles; red = activated glia, purple = ROS. (TIF) [file pone.0073631.s004.tif]

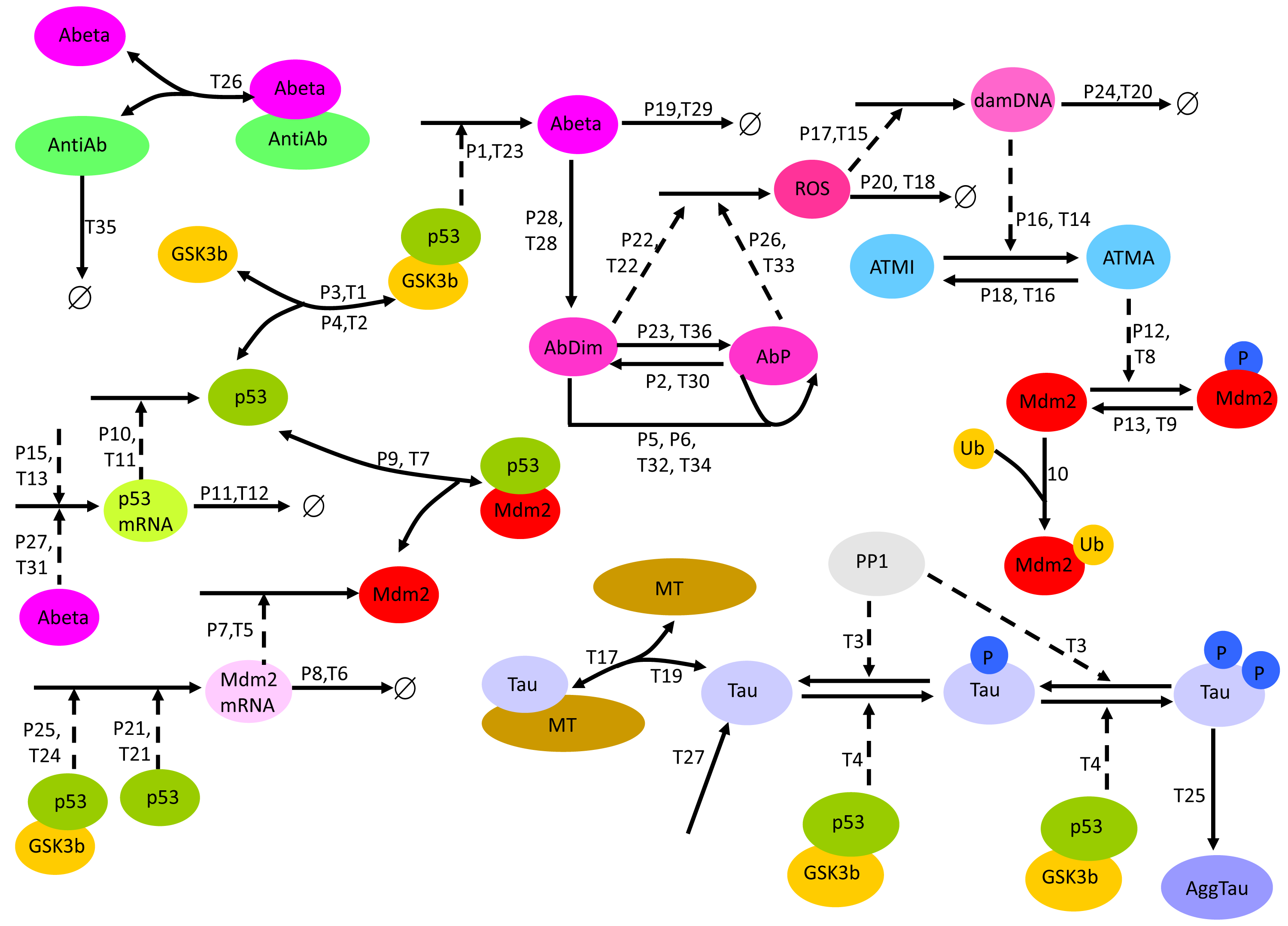

Supplement: Figure S5 — Key components of the model network. Network diagram of the key components in the model network showing the reactions involving the most sensitive parameters. The labels on the reaction arrows starting with ‘P’ or ‘T’ indicate the rank of the parameter with respect to its effect on the maximum level of plaques and tangles respectively. (TIF) [file pone.0073631.s005.tif]
